# Experienced complaints, activity limitations and loss of motor capacities in patients with pure hereditary spastic paraplegia: a web-based survey in the Netherlands

**DOI:** 10.1186/s13023-020-1338-4

**Published:** 2020-03-04

**Authors:** Bas J. H. van Lith, Hans C. J. W. Kerstens, Laura A. C. van den Bemd, Maria W. G. Nijhuis-van der Sanden, Vivian Weerdesteyn, Rob J. E. M. Smeets, Klemens Fheodoroff, Bart P. C. van de Warrenburg, Alexander C. H. Geurts

**Affiliations:** 1 Department of Rehabilitation, Donders Institute for Brain, Cognition and Behaviour, Radboud university medical center, PO Box 9101, 6500 HB Nijmegen, The Netherlands; 2 HAN University of Applied Sciences, PO Box 6960, 6503 GL Nijmegen, The Netherlands; 3 Radboud Institute for Health Sciences, Scientific Institute for Quality of Healthcare, Radboud university medical center, PO Box 9101, 6500 HB Nijmegen, The Netherlands; 4 Department of Rehabilitation, Sint Maartenskliniek, PO Box 9011, 6500 GM Nijmegen, The Netherlands; 5 Department of Rehabilitation Medicine, Maastricht University, Research School CAPHRI, PO Box 616, 6200 MD Maastricht, the Netherlands; 6 Gailtal-Klinik, Radnigerstrasse 12, 9620 Hermagor, Austria; 7 Department of Neurology, Donders Institute for Brain, Cognition and Behaviour, Radboud university medical center, PO Box 9101, 6500 HB Nijmegen, The Netherlands

**Keywords:** Hereditary spastic paraplegia, Spastic paraparesis, Survey, Falls, Gait, Rehabilitation

## Abstract

**Background:**

Hereditary spastic paraplegia (HSP) is a group of inherited disorders characterized by progressive spastic paresis of the lower limbs. Treatment is often focused on reducing spasticity and its physical consequences. To better address individual patients’ needs, we investigated a broad range of experienced complaints, activity limitations, and loss of motor capacities in pure HSP. In addition, we aimed to identify patient characteristics that are associated with increased fall risk and/or reduced walking capacity.

**Methods:**

We developed and distributed an HSP-specific online questionnaire in the Netherlands. A total of 109 out of 166 questionnaires returned by participants with pure HSP were analyzed.

**Results:**

Participants experienced the greatest burden from muscle stiffness and limited standing and walking activities, while 72% reported leg and/or back pain. Thirty-five and 46% reported to use walking aids (e.g. crutches) indoors and outdoors, respectively; 57% reported a fall incidence of at least twice a year (‘fallers’); in 51% a fall had led to an injury at least once; and 73% reported fear of falling. Duration of spasticity and incapacity to rise from the floor were positively associated with being a ‘faller’, whereas non-neurological comorbidity and wheelchair use were negatively associated. Higher age, experienced gait problems, not being able to stand for 10 min, and incapacity to open a heavy door showed a negative association with being a ‘walker without aids’ (> 500 m).

**Conclusions:**

Our results emphasize the large impact of spastic paraparesis on the lives of people with pure HSP and contribute to a better understanding of possible targets for rehabilitation.

## Background

Hereditary spastic paraplegia (HSP) is a group of inherited disorders, characterized by progressive bilateral lower limb spasticity and, to a lesser extent, muscle weakness [1]. HSP can be classified as ‘pure’ (‘uncomplicated’) or ‘complicated’, depending on the presence of other neurologic abnormalities such as ataxia, seizures, cognitive impairment, and/or involvement of the upper extremities and speech [2–5]. In patients with pure HSP, the main neurological feature is a progressive spastic paraparesis (SP). As HSP cannot be cured, treatment is often focused on reducing or stabilizing spasticity and its physical consequences. However, rehabilitation strategies should focus on a broad range of experienced complaints and limitations to address the needs of patients. Thus, gaining more knowledge of SP-related complaints, activity limitations, and loss of motor capacities as experienced by patients is important for better disease management and tailoring interventions to individual patients’ needs.

In various patient groups with lower limb spasticity (e.g. stroke, spinal cord injury and multiple sclerosis), spasticity appears to be a significant contributor to experienced complaints, activity limitations, and loss of motor capacities [6–8]. Recently, an international survey in patients living with spasticity was conducted that emphasized the large impact of spasticity on daily life and the need for better collaboration, communication and sharing of information between patients and their healthcare providers to fulfill individual needs [9]. Yet, patients with pure HSP may differ from the population with spastic paraparesis at large, as their condition is inherited and slowly progressive. Existing reports on pure HSP typically indicate the presence of gait and balance impairments and an increased risk of falling as the most prominent functional consequences [5, 10–14]. In addition, some studies mentioned the occurrence of pain, fatigue, urinary symptoms, sleeping problems, unpredictable day-to-day fluctuations, activity limitations and participation restrictions. However, these studies included either small patients samples [15, 16] or lumped patients with pure and complex HSP [17–19].

Against this background, we developed a disease-specific online questionnaire to investigate the experienced complaints, activity limitations, and loss of motor capacities as well as the experienced healthcare needs in a large, representative group of patients with pure HSP in the Netherlands. In the current study, we specifically focus on SP-related complaints, activity limitations and loss of motor capacities to better understand the impact of the disease. Besides motor problems, we included autonomic (micturation, defecation, and sexual) dysfunctions, as we expected that the latter might be related to spasticity as well. In addition, as balance and gait impairments are considered key problems in HSP, we aimed to identify specific demographic, clinical and functional characteristics that are associated with increased fall risk and/or reduced walking capacity. Data on the experienced needs will be reported in a separate publication.

## Methods

### Recruitment and inclusion of participants

Participants were recruited through the national patient organization for neuromuscular disorders in the Netherlands (“Spierziekten Nederland”; www.spierziekten.nl). On our request, they sent all the members of the HSP working group an e-mail with information about the web-based survey. In addition, all patients with pure HSP known at the national expertise center for inherited movement disorders of the Radboud university medical center in Nijmegen were sent a letter with information about the survey. People could participate if they had genetically confirmed HSP, or were very likely to have HSP based on their clinical symptoms and family history. In addition, participants had to be 18 years or older. Persons with HSP and their relatives were requested to contact the primary researcher (BvL) by e-mail if they were willing to participate. After receiving an e-mail, the primary researcher sent a unique link to the web-based questionnaire to each patient who had indicated willingness to participate. This study was approved by the regional medical ethics committee “Commissie Mensgebonden Onderzoek Arnhem-Nijmegen” (number 2016–2922) and conducted according to the declaration of Helsinki.

### Web-based questionnaire

The structure and content of the web-based survey were designed by a team of expert physicians, researchers, physical therapists, and persons with HSP. Part of the questionnaire was based on a previous international survey of patients living with spasticity [9], while other questions were based on a qualitative study in patients with pure HSP who were interviewed about the daily life consequences of spastic paraparesis and their related healthcare needs (note: data on healthcare needs are reported elsewhere) [16]. The structure and formulation of the questions and predefined answering options were improved during an iterative process, until all authors agreed on the final questionnaire. Completion of the questionnaire by participants was estimated to take about 20 min, but there was no set time limit. Participants were able to pause the questionnaire and continue later. To some extent, the amount of questions was variable for each participant, depending on his/her answer to a preceding question. Answering options were based on multiple choice, but some questions included a text entry as one of the options. Overall, the questions in this study were grouped into three response categories: A. ‘participant characteristics’, B. ‘complaints and activity limitations’, and C. ‘loss of motor capacities’ (see Appendix). Whereas the questions in category B were focused on the problems participants experienced when performing certain daily life activities, the questions in category C were focused on the self-rated capacity to execute specific activities. To obtain a homogeneous sample of persons with pure HSP, specific questions were included to identify patients with neurological comorbidity and/or a complicated form of HSP.

### Data analysis

Patients were excluded from further analysis if they indicated that they had a complicated form of HSP (or a genetic defect invariably associated with a complicated form of HSP); experienced upper limb paresis, speech problems, or cognitive problems; or reported any neurological comorbidity that could influence spasticity, motor control, physical fitness, or activity. As we were interested in SP-related complaints, activity limitations, and loss of motor capacities, participants who reported that they did not experience spasticity (or had spasticity for less than 1 year) were also excluded from further analysis.

### Statistical analysis

Descriptive statistics were used to analyze the primary data obtained from the questionnaires. In addition, univariate logistic regression analyses were performed on two dependent variables: being a ‘walker without aids’ (i.e., self-report of walking distance without crutch or walker > 500 m), and being a ‘faller’ (i.e., self-report of at least two falls a year). The threshold of at least two falls a year was chosen to make sure that subjects were not classified as a ‘faller’ based on a single (perhaps coincidental) fall. To prevent overfitting of the model in case of many correlations between possible determinants and dependent variables, we continued with multivariate logistic *forward* regression analyses. Thus, possible determinants from each of the three response categories that were associated with a specific dependent variable in the univariate analyses (*p* < 0.10) were entered in a multivariate logistic forward regression analysis (*p* < 0.05) to identify clinically relevant, independent determinants for this dependent variable.

## Results

### A. Participant characteristics

A total of 194 respondents requested to participate, of which 166 persons returned a fully completed questionnaire. Subsequently, 57 respondents not meeting the criteria outlined above were excluded (*n* = 16 established complicated form of HSP; *n* = 12 upper limb paresis; *n* = 21 speech problems; *n* = 19 cognitive problems; *n* = 15 neurological comorbidities; *n* = 4 spasticity < 1 year). Finally, 109 respondents were included for further analysis. The participants showed an equal sex distribution (49.5% male and 50.5% female) and had a mean age of 52.8 years (age range 19–84 years). Most participants (83%) indicated a positive family history for pure HSP and 53% reported that HSP was also genetically confirmed. Participants without a positive family history or genetic diagnosis (*n* = 7) were e-mailed by the primary investigator to confirm that the clinical diagnosis of pure HSP was made by a neurologist. All participant characteristics are summarized in Table 1.

**Table 1:** Demographic and clinical characteristics

	*N* = 109	
Patient characteristics	*n*	mean (SD)
Sex (male/female)	54/55	
Age (years)		52.8 (14.2)
Duration of spasticity symptoms (years)		
*1–5 years*	21	
*6–10 years*	21	
*11–15 years*	16	
*> 15 years*	51	
Genetic defect	57	
*SPG-3a*	4	
*SPG-4*	36	
*SPG-5a*	2	
*SPG-7*	3	
*SPG-8*	3	
*SPG-10*	2	
*SPG-17*	1	
*SPG-31*	5	
*SPG-72*	1	
Positive family history		
*First degree relatives*	83	
*Other family members*	47	
*First degree relatives and/or other family members*	90	
*Unknown*	8	
*No*	11	
Non-neurological comorbidities		
*Asthma/COPD*	6	
*Diabetes*	1	
*Hypertension*	9	
Joint disorders*	13	
*Cardiac problems*	4	
*Other non-neurological comorbidities*	7	

*Rheumatism and osteoarthritis were given as examples to participants. Patients were instructed that this category did not include non-specific back complaints

### B. Complaints and activity limitations

**Fig. 1:**
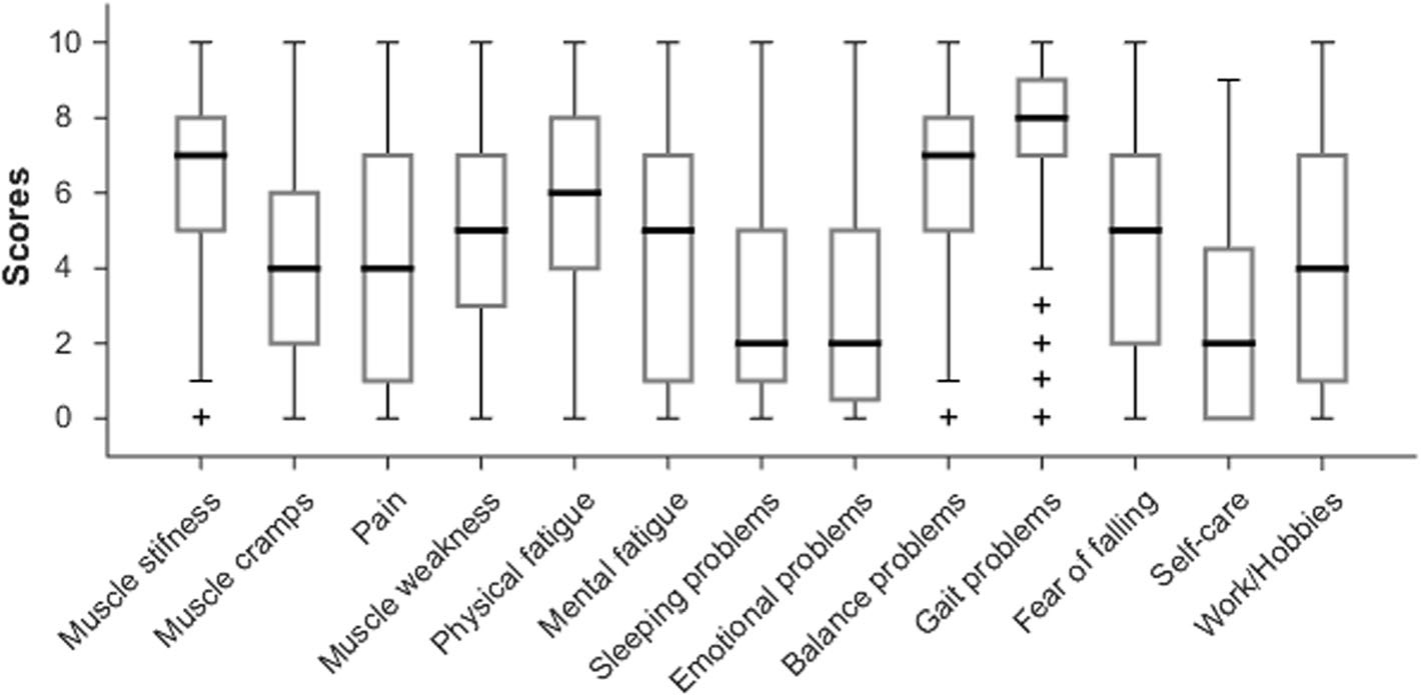
Median, interquartile range, and total range of the level of burden/hindrance that participants experienced in various categories (Questions B1-B13) (0: no burden/hindrance, 10: extreme burden/hindrance). +: outlier

Experienced complaints and activity limitations were scored on a numeric scale (range 0–10; 0: no burden/hindrance, 10: extreme burden/hindrance). Overall, the participants experienced the greatest burden or hindrance from their muscle stiffness and from problematic standing and walking activities (Fig. 1). They also reported to experience a substantial burden from both physical and mental fatigue. Sleeping problems, self-care problems, and emotional problems were relatively mild.

Seventy-two percent of the participants reported pain. Fifty-five participants (50%) reported back pain, predominantly in the lower back, and 59 participants (54%) reported leg pain. The majority described leg pain as nerve pain (i.e., burning or tingling pain; *n* = 25), cramps (*n* = 32), or restless legs (i.e., strong urge to keep the legs moving; *n* = 33).

#### Autonomic dysfunctions

Forty-nine (50%) and 21 (19%) of the participants reported micturation and defecation problems, respectively. These problems were related to extreme urge (*n* = 22 and *n* = 11, respectively), impaired sphincter control (*n* = 45 and *n* = 14, respectively), or slowness of gait (*n* = 30 and n = 11, respectively). Forty-three of the participants (39%) reported sexual problems, whereas 46 (42%) experienced no sexual problems and 20 (18%) did not know. The most frequently reported reasons for sexual problems were related to spasticity (*n* = 26) and pain (n = 14). Some men reported ejaculation problems (*n* = 4) or erectile dysfunction (*n* = 8), whereas some women experienced vaginal dryness (*n* = 5). Eleven participants reported other sexual problems, among which difficulties with having an orgasm (*n* = 6).

### C. Loss of motor capacities

Table 2 provides an overview of the devices applied for supporting functional mobility. The devices were categorized into orthoses (including orthopedic footwear), walking aids, and wheelchairs. The most often used devices were walking aids, such as canes or crutches: 35% reported to use walking aids indoors, whereas 46% used walking aids outdoors. Outdoors, an (electric) wheelchair was often applied as an alternative for walking.

**Table 2:** Use of mobility-supporting devices (N = 109)

	n (%)	
Type of device	Indoors	Outdoors
Orthoses	32 (29%)	44 (40%)
*Ankle-foot orthosis*	13 (12%)	19 (17%)
*Orthopedic footwear*	28 (26%)	34 (31%)
Walking aids	38 (35%)	50 (46%)
*Walker*	26 (24%)	30 (28%)
*Cane/Crutch*	28 (26%)	41 (38%)
Wheelchairs	23 (21%)	50 (46%)
*Wheelchair*	22 (20%)	43 (39%)
*Electric wheelchair*	7 (6%)	29 (27%)
Other	11 (10%)	10 (9%)
None	44 (40%)	23 (21%)

**Fig. 2:**
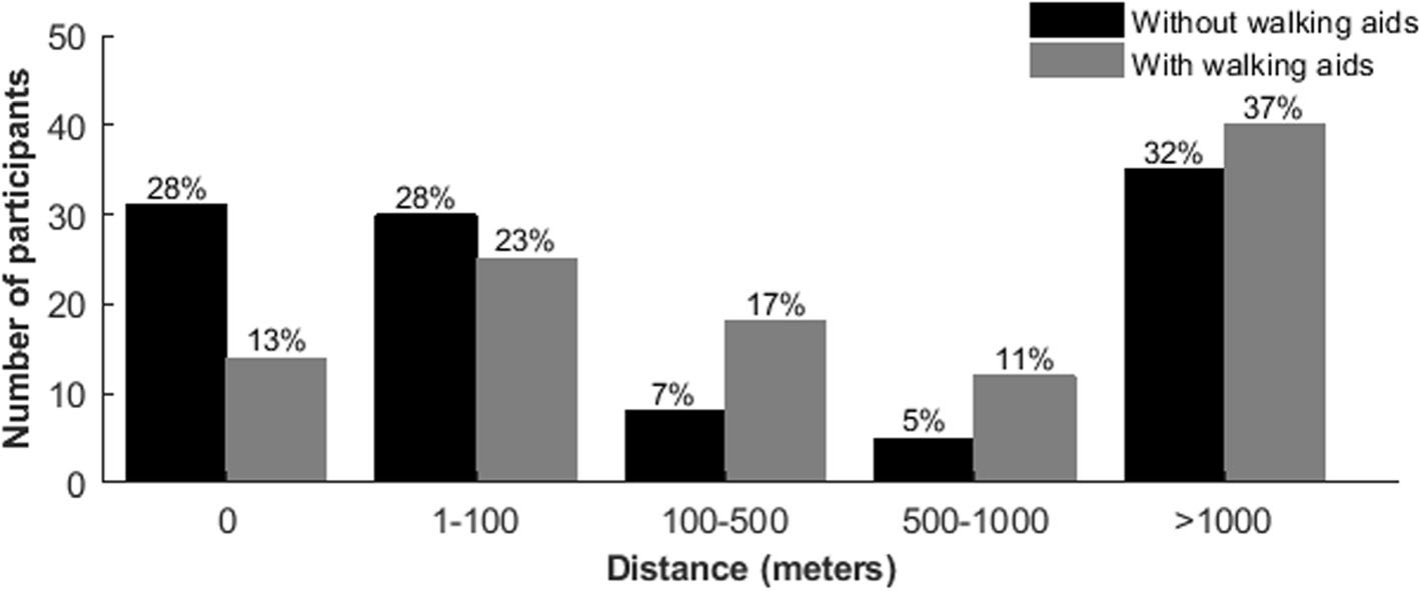
Walking distance with and without aids

Without a walking aid, the majority of the participants was either unable to walk (28%) or walk up to 100 m (28%), or was able to walk more than 1000 m (32%). Only 12% reported to be able to walk between 100 and 1000 m without a walking aid. With the use of walking aids, there was a more equal distribution of walking capacity due to a general shift from very short to longer distances (Fig. 2). Indeed, a total of 45 participants (41%) was able to walk further with than without the use of a walking aid. Many participants described their gait pattern as characterized by drop foot (75%), scissoring (30%), and/or crouch gait (13%).

**Fig. 3:**
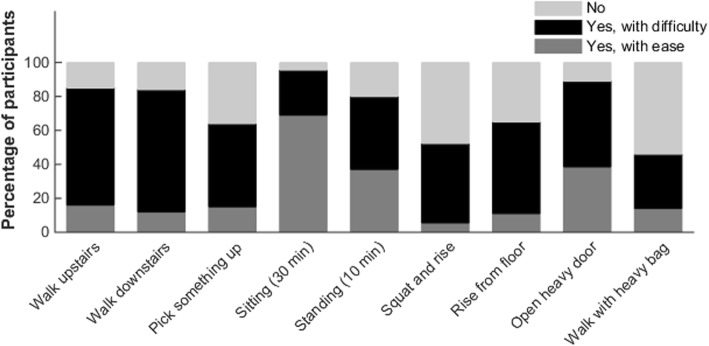
Percentage of participants that responded to be able to execute specific motor capacities with ease, with difficulty, or not at all

As shown in Fig. 3, relatively few participants were not able to sit for 30 min or to open a (heavy) door. In contrast, stair walking, picking something up from the floor, squatting and rising, rising from the floor, and walking with a heavy bag was difficult or even impossible for the majority of the participants.

#### Falls

**Fig. 4:**
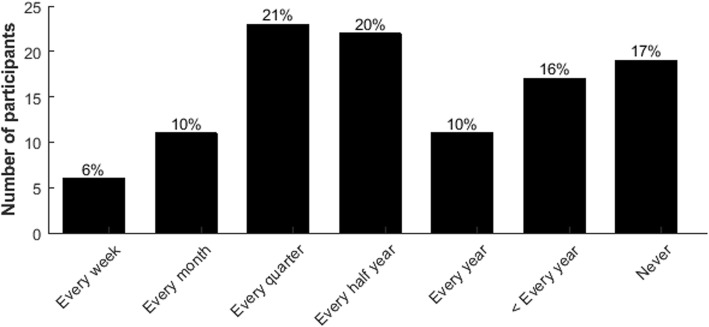
Self-reported fall frequency of participants

Since the onset of spasticity symptoms, 93 of the participants (85%) had fallen at least once, which had occurred within 5 years of symptom onset in 63 of 93 subjects (68%). Most participants (67%) reported a fall incidence of at least once a year; 57% reported to fall at least twice a year (‘faller’) (Fig. 4). In 56 participants (51%) a fall had led to an injury at least once, such as a skin injury (*n* = 34), bruise (*n* = 33), and/or bone fracture (*n* = 13). Only 29 of the participants (27%) reported not to be afraid of falling, whereas 66 (60%) were moderately afraid and 14 (13%) were very afraid to fall.

### Regression analyses

Univariate logistic regression analyses (*n* = 109) revealed nine determinants that were associated with being a ‘faller’. These determinants were entered into a multivariate forward regression analysis (Table 3, upper part). In the final model, duration of spasticity symptoms and the incapacity to rise from the floor were positively associated with being a faller, whereas non-neurological comorbidities and the use of a wheelchair were negatively associated. The overall explained variance was 45%.

**Table 3:** Multivariate forward logistic regression analyses

Dependent variable: ‘faller’ (self-report of at least two falls a year) (*n* = 109)				
Cofactor	Level of cofactor	n	OR (95% CI)	*p*-value
Duration of spasticity symptoms	*Reference:* 1–5 years	21		
	5–10 years	21	39.070 (5.405–282.410)	< 0.001
	10–15 years	16	7.789 (1.419–42.757)	0.018
	> 15 years	51	18.025 (3.813–85.220)	< 0.001
Comorbidity	*Reference:* No	101		
	Yes	8	0.162 (0.052–0.509)	0.002
Walking aids indoors	*Reference:* No wheelchair	87		
	Wheelchair	22	0.130 (0.029–0.577)	0.007
Rise from floor	*Reference:* Yes, with ease	12		
	Yes, with difficulty	59	17.897 2.759–116.108)	0.002
	No	38	17.934 (2.333–137.878)	0.006
Overall explained variance R^2^ = 0.450				
Dependent variable: ‘walker without aids’ (self-report of walking distance without crutch or walker > 500 m) (n = 82)				
Cofactor	Level of cofactor	n	OR (95% CI)	*p*-value
Age	19–84	82	0.944 (0.899–0.994)	0.030
Gait problems	0–10	82	0.604 (0.400–0.911)	0.016
Standing (10 min)	*Reference:* Yes, with ease	40		
	Yes, with difficulty	42	0.286 (0.085–0.970)	0.045
Open heavy door	*Reference:* Yes, with ease	41		
	Yes, with difficulty	41	0.165 (0.049–0.564)	0.004
Overall explained variance R^2^ = 0.583				

The 27 participants who were not able to stand for 10 min or who were not able to open a heavy door, were never able to walk more than 500 m without walking aids. These participants could not be included in the (multivariate) logistic regression, because of the 1:1 associations. Univariate logistic regression analyses of the remaining participants (*n* = 82) yielded 29 determinants that were associated with being a ‘walker without aids’. These determinants were entered into a multivariate forward logistic regression analysis (Table 3, lower part). In the final model, difficulties with standing for 10 min and with opening a heavy door showed a strong negative association with being a ‘walker without aids’, whereas age and experienced gait problems showed a small to moderate negative association, respectively. The overall explained variance was 58%.

## Discussion

Given the estimated prevalence of 800 persons with pure HSP in the Netherlands [20], this web-based survey probably included a fairly representative sample of 109 persons who showed an equal sex distribution, a wide age range, a large variation in duration of spasticity symptoms, and an expected (skewed) distribution of underlying genetic defects. This study sample reported many subjective complaints and activity limitations, of which muscle stiffness and problems with performing standing and walking activities were most severe (medians ≥7 on a numeric rating scale 0–10).

### Balance, gait and falls

Several studies have reported problems with performing standing and walking activities and an increased fall risk in patients with HSP [4, 11–14], but the published data on severity or prevalence of these problems are still very limited. Only one survey reported that 47% of the participants with HSP had fallen at least once over the past three months [18], yet this estimate also included patients with complicated forms of HSP. In the current survey, both the reported severity of the balance and gait problems, fear of falling, and the high prevalence of falls and fall-related injuries indicate that safe and efficient postural and ambulatory control is a major problem in people with pure HSP. The observed proportion of 67% of people who reported at least one fall per year seems to be comparable to other patients with spastic paraparesis caused by, for instance, multiple sclerosis, tropical spastic paraparesis or spinal cord injury of whom 50–75% report at least one fall per year [21–30].

Unfortunately, the present data do not allow us to make inferences on the effect of walking aids on falls, but many participants reported the use of different types of walking aids to increase their walking distance. Without a walking aid, 63% indicated that they could not walk at least 500 m, while 28% were not able to walk at all. Besides walking aids, several participants used some type of ankle-foot orthosis or orthopedic footwear to improve their walking capacity, probably to prevent foot drag during the swing phase and/or optimize ankle stability during the stance phase. Based on the results of this study, it is not possible to conclude which walking aid and/or orthosis is generally most effective. Our experience has learned that a thorough individual clinical assessment, sometimes supported by an instrumented gait analysis, is the best way to provide an individually tailored advice for the use of medical devices. This advice should take into account both the gait pattern (e.g., foot drag, crouch, scissoring) and the execution of other daily life activities than upright standing and walking, such as stair climbing, squatting, cycling, driving a car etc. [13]. Several interventional studies have provided indications of improved gait capacity by robotic training [31], botulinum toxin injections in spastic calf muscles or hip adductors [32–35], and intrathecal baclofen [36]. However, these studies mainly focused on gait speed and/or gait pattern as outcomes, and not on walking distance, performance of daily life activities, or falls. Studies on surgical interventions in HSP have not been conducted yet. Overall, it is fair to conclude that there is an urgent need for future studies in people with HSP that investigate the underlying mechanisms of their balance and gait problems and increased fall risk in order to develop novel and convenient intervention strategies to preserve life-long ambulatory capacity and gait-related activities, and prevent falls. Remarkably, even sitting for 30 min appeared to be a problem for 30% of the participants, perhaps due to back pain or severe stiffness. Further research on sitting problems is necessary, particularly regarding wheelchair mobility.

### Muscular and non-motor symptoms

Unsurprisingly, muscle stiffness, muscle cramps and weakness appeared to be significant problems in our participants. Usually stiffness and cramps are treated with muscle relaxant medication, but apparently these symptoms are still very troublesome for many patients. Our results further showed that pain, fatigue, and autonomic problems are major (non-motor) symptoms in patients with pure HSP. Only few previous studies have mentioned pain as an important problem in this population [15, 16, 18, 37], even though pain in the legs and/or back was reported by 72% of our participants. This number was similar to the reported frequency in previous studies [15, 18]. The nature of leg pain was most often described as nerve pain, cramping pain or restless legs. Back pain was most prevalent in the *lower* back. In our clinical experience, low back pain often has a continuous character, possibly related to postural deviation (i.e., anterior pelvic tilt with lumbar hyperlordosis). On average, the severity of pain yielded a median score of 4 on a numeric rating scale (0–10), but from a recent qualitative study, using semi-structured interviews in 14 patients with pure HSP, we learned that, in individual patients, pain may be severe enough to seriously affect their quality of life [16]. Besides pain, many participants experienced fatigue, both physically and mentally, with median severity scores of 6 and 5, respectively. From our recent qualitative study it became clear that spasticity and muscle stiffness impact on physical fitness, while the high levels of attention needed to cope with balance and gait problems seem to cause mental fatigue [16]. Generally, fatigue and pain are serious problems in many types of chronic neurological disorders such as spinal cord injury, stroke, Parkinson’s disease, multiple sclerosis, and neuromuscular disease, which require specific clinical attention and treatment [30, 38–42]. Our results indicate that people with HSP form no exception to this rule, and probably remain undertreated in these respects. The present results confirm previously described micturation and defecation problems in people with pure HSP [43, 44]. As underlying causes for these problems our data indicate, on the one hand, extreme urge and problematic sphincter control [19] and, on the other hand, problems to reach the toilet in time. The latter problems may well be primarily related to the gait disorder due to the spastic paraparesis. Whereas urological consultation is needed in the case of bladder and sphincter abnormalities, adequate treatment of spasticity and gait problems may be additionally helpful to reduce micturation and defecation problems. Sexual problems were also frequently mentioned by our participants. This result confirms a previous study reporting sexual complaints in 7 out of 11 patients with pure HSP, indicating pain and spasticity as most important underlying causes [44]. Hence, also for sexual problems, a combination of urological / gynecological consultation and adequate spasticity management seems to be crucial.

### Predicting unsupported walking capacity and falling

The duration of spasticity symptoms appeared to be a strong predictor of being a faller. However, unexpectedly, people with an intermediate duration of spasticity symptoms (6–10 years) showed the highest risk, much higher than those with a shorter duration (1–5 years), and also higher than those with a duration of more than 15 years. A possible explanation for this finding may be that in this stage of the disease (6–10 years) people are becoming increasingly affected by spastic paraparesis, while they are still trying to remain as active as possible in terms of standing and walking activities. This discrepancy might bring about an increased fall risk. In the next phase (11–15 years symptom duration), there seems to be a marked drop in fall risk, which might be related to a gradual adjustment of the activity pattern, increased carefulness, and perhaps the use of walking aids. Symptom duration longer than 15 years seems to increase fall risk again, perhaps due to the severity of the balance problems, affecting basic activities such as rising to stance, sitting down, and making transfers. Unfortunately, our data do not allow more detailed interpretation of the observed risk pattern, which warrants further investigation. Difficulty or inability to rise from the floor was another important risk factor of being a faller, which is an intuitive finding, as rising from the floor requires both sufficient lower extremity strength and basic balance capacity. Testing the individual capacity to independently rise from the floor may, therefore, be an interesting clinical test to assess increased fall risk. The use of a wheelchair and the presence of non-neurological comorbidities appeared to be strong ‘protectors’ against being a faller. The remarkable result of non-neurological comorbidities being negatively associated with falling may be related to a lower level of standing and walking activities in these people, which may lead to a reduced fall risk. However, further research should shed more light on these findings.

With regard to walking, higher age was associated with a lower chance of being able to walk without aids for at least 500 m, which probably results from a combination of normal ageing and disease progression. The observed odds ratio of 0.944 implies that, with every year, the chance of being a walker without walking aids decreases by a factor 0.944. Over 30 years, the chance of being a walker without aids would thus decrease to a mere 17.8% (0.944^30^). Experienced problems with gait-related activities also appeared to be a strong predictor of the inability to walk without aids. Lastly, the inability (or difficulty) to stand for 10 min and/or open a heavy door were also very strong predictors. This points towards the notion that standing balance capacity is an important prerequisite for independent walking. Asking patients in clinical practice whether they can easily stand for 10 min and/or open a heavy door may, thus, provide a good indication of their independent walking capacity. Again, further research should provide more insight in these relationships.

### Strengths and limitations

A limitation inherent in using questionnaires is the subjective nature of the results and the lack of physical examination data, which is why we emphasized that our data reflect the *experienced* complaints, activity limitations, and loss of motor capacities in people with HSP. Still, experiences may reveal problems that remain unnoticed when focusing on objective assessments. In addition, the design process of the questionnaire was not unbiased, as it was based on professionals’ preconception of what important aspects of HSP might be. Yet, although participants often had the option to provide additional information next to the predefined multiple choice options, they used this option only sparsely.

Another limitation is that only the subjects without a positive family history or genetic diagnosis were checked for a formal diagnosis of pure HSP made by a neurologist. By including and excluding subjects based on specific questions, we tried to obtain a homogeneous sample with pure HSP, but it is possible that some people were incorrectly enrolled or excluded. Our study sample was limited in absolute numbers, which precluded subgroup analyses based on for instance genetic defect, or duration of spasticity symptoms. Nevertheless, our study is the largest survey in people with pure HSP until now. In addition, our participants showed an equal sex distribution, wide age range, and a large variation in SP-related symptoms, which supports their representativeness of the Dutch population with pure HSP, of which we probably included about 15% [20]. Since we excluded patients with complicated forms of HSP, our results cannot be generalized to the entire population with HSP.

## Conclusion

The results of this web-based survey indicate that people with pure HSP experience many physical complaints, activity limitations, and loss of motor capacities. Of these, muscle stiffness, limited standing and walking activities, and increased fall risk are most prominent, but also pain, fatigue, autonomic dysfunctions, fear of falling, and problems with performing working and hobby activities are relevant symptoms and/or areas of disability with an inherent loss of quality of life. Future research, using objective next to subjective measures, is needed to better understand the full functional impact of HSP on the daily lives of patients, to study underlying mechanisms of disabling symptoms, and to find new roads to interventions that are able to preserve balance and ambulatory capacity as well as limit the burden of the non-motor symptoms.

## Supplementary information

**Additional file 1.** Appendix 1. Overview of variables and answers.

## Data Availability

The dataset supporting the conclusions of this article is included within the article and its additional files.

## Abbreviations

HSP: Hereditary spastic paraplegia
SP: Spastic paraparesis
